# Parental Knowledge, Self-confidence, and Usability Evaluation of a Web-Based Infographic for Pediatric Concussion: Multimethod Study

**DOI:** 10.2196/36317

**Published:** 2022-05-10

**Authors:** Alyson Campbell, Lisa Hartling, Vickie Plourde, Shannon D Scott

**Affiliations:** 1 Faculty of Nursing University of Alberta Edmonton, AB Canada; 2 Department of Pediatrics Faculty of Medicine and Dentistry University of Alberta Edmonton, AB Canada; 3 École de Psychologie Faculté des sciences de la santé et des services communautaires Université de Moncton Moncton, NB Canada

**Keywords:** concussion, mTBI, usability evaluation, knowledge assessment, knowledge translation, parent knowledge, parent confidence, patient engagement, educational tool

## Abstract

**Background:**

Concussions, which are known as mild traumatic brain injuries, are complex injuries caused by direct or indirect blows to the head and are increasingly being recognized as a significant public health concern for children and their families. Previous research has identified few studies examining the efficacy of educational interventions on parental concussion knowledge. The aim of this research was to actively work together with children who have experienced a concussion and their parents to develop, refine, and evaluate the usability of a web-based infographic for pediatric concussion.

**Objective:**

The objective of this study was to report on the usability of the infographic, parental knowledge, and self-confidence in pediatric concussion knowledge before and after exposure to the infographic.

**Methods:**

A multiphase, multimethod research design using patient engagement techniques was used to develop a web-based infographic. For this phase of the research (usability, knowledge, and confidence evaluation), parents who could communicate in English were recruited via social media platforms and invited to complete web-based questionnaires. Electronic preintervention and postintervention questionnaires were administered to parents to assess changes to concussion knowledge and confidence after viewing the infographic. A usability questionnaire with 11 items was also completed.

**Results:**

A web-based, infographic was developed. The infographic is intended for parents and children and incorporates information that parents and children identified as both wants and needs about concussion alongside the best available research evidence on pediatric concussion. A total of 31 surveys were completed by parents. The mean scores for each item on the usability surveys ranged from 8.03 (SD 1.70) to 9.26 (SD 1.09) on a 10-point Likert scale, indicating that the usability components of the infographic were largely positive. There was no statistically significant difference between preintervention and postintervention knowledge scores (*Z*=−0.593; *P*=.55; both preintervention and postintervention knowledge scores had a median of 9 out of 10). In contrast, there was a statistically significant difference between preintervention (mean 3.9/5, SD 0.56) and postintervention (mean 4.4/5, SD 0.44) confidence in knowledge scores (t_30_=−5.083; *P*<.001).

**Conclusions:**

Our results demonstrate that parents positively rated a web-based, infographic for pediatric concussion. In addition, although there was no statistically significant difference overall in parents’ knowledge scores before and after viewing the infographic, their confidence in their knowledge did significantly increase. These results suggest that using a web-based infographic as a knowledge translation intervention may be useful in increasing parents’ confidence in managing their child’s concussion.

## Introduction

### Background

Concussions, which are known as mild traumatic brain injuries, are complex injuries caused by direct or indirect blows to the head [1]. An estimated 125,000 youths in Canada and 750,000 in the United States will sustain concussions annually [2,3]. Typical signs and symptoms of a concussion include headaches, nausea, dizziness (or fogginess), and sensitivity to light or noise [4]. For most children, recovery occurs within 1 to 4 weeks after the injury [5]. An estimated one-third of children and youths will have persistent symptoms that occur beyond 28 days. Persistent symptoms may include headaches, sleep problems, and emotional distress [2,3].

Rapid increases in the amount of health research, particularly in pediatric concussion, and the increased accessibility to research offered through the internet, suggest a demand for reliable, evidence-based health information that is relevant and easy to understand for patients and families [6]. Innovative media have been shown to be superior to traditional methods (ie, handouts) for transferring health information to consumers [7]. Our research team conducted an environmental scan of pediatric concussion resources found on the internet and in app stores. Despite innovative media being superior to traditional methods for transferring health information, our scan revealed that most pediatric concussion resources are PDF documents, suggesting that organizations struggle to optimize the use of innovative media (eg, infographics, videos, and narratives) when sharing health information. Our environmental scan also revealed that many resources are not developed in collaboration with health consumers. Using patient engagement approaches to involve end users (eg, parents and children) in the development of educational resources is key to effective knowledge translation (KT) [8].

A systematic review [9] evaluating the effectiveness of concussion education programs for coaches and parents of youth athletes found a limited number of studies examining the efficacy of educational interventions on parental knowledge of concussion and a lack of interventions designed specifically for parents. This suggests that additional research is necessary to investigate changes in parental knowledge following educational interventions and a need for more interventions specifically intended for parents and families. Furthermore, although studies have assessed parental knowledge of concussion, most have focused on sport-related concussions [9-14]. Few studies have evaluated changes in concussion knowledge after exposure to an educational intervention [13], and no known studies have assessed changes in self-confidence. Confidence is an important construct for behavior change. Higher levels of confidence increase the likelihood that an individual will change a health behavior when faced with obstacles [15]. Understanding how confident parents are in their knowledge and abilities to parent is an essential component of the quality and sustainability of parenting behaviors [16]. In the context of our research, understanding how confident parents are in their knowledge of concussion and if an increase in confidence before and after an intervention is observed may provide critical insight into their health decision-making for their child, such as when to seek emergency care and at-home recovery.

To date, KT efforts have largely focused on ensuring that health care professionals use the latest research to inform their practice; however, emerging evidence suggests that efforts that target health consumers (eg, patients and families) can inform their decision-making and shape their treatment expectations (eg, what to expect in the emergency department) [17-20]. Although research is beginning to demonstrate that strategies targeting parents for KT can reduce health care use and improve outcomes, more research is required to fully understand the power and impact of these efforts on both children and families [21-23].

### Objectives

Developing innovative KT tools that present research-based information in user-friendly languages and formats provides consumers with accurate recommendations while addressing knowledge or information needs. In addition, these tools may foster and empower consumers to make informed decisions about health care for themselves and their families. To date, limited research has explored the usefulness and effectiveness of web-based infographics as an innovative way to share health information with patients and families. The purpose of this research was to actively work together with children who have experienced a concussion and their parents to develop, refine, and evaluate the usability of a web-based infographic for pediatric concussion. We also aimed to assess parental self-confidence and knowledge of pediatric concussion before and after exposure to the infographic. This paper provides an overview of the development of the infographic prototype and reports on the results of parental usability, knowledge, and confidence testing.

## Methods

### Overview

A multiphase, multimethod study using patient engagement techniques was used to develop, refine, and evaluate the usability of a web-based infographic for pediatric concussion to promote KT. Details of phases 1 and 2 of this work are reported by Campbell et al [24]. Changes in concussion knowledge and confidence in responses were also evaluated through preintervention and postintervention tests.

### Ethics Approval

Ethics approval was obtained from the University of Alberta Health Research Ethics Board (Pro0096202).

### Exploring Gaps in Current Concussion Tools (Intervention Development, Phase 1)

An environmental scan was conducted (May 2021) by the first author (AC) to identify publicly available Canadian developed resources providing information on pediatric concussion found on the internet and in apps. Information gaps in these resources were extracted (eg, resource format, target audience, and information) and used to inform the subsequent phases of this research, including the target audience for semistructured interviews (phase 2) and elements of the infographic, including target audience, content, and format (phase 3). The full results of this environmental scan are reported by Campbell et al [24].

### Compilation of Child and Parent Narratives (Intervention Development, Phase 2)

Phase 2 of this multiphase study was a qualitative description [25,26]. The first author (AC; trained in qualitative methods and supervised by the senior author and principal investigator (SDS) conducted semistructured interviews with a convenience sample of children who have experienced a concussion and their parents. Children and parents were recruited on the web through our research group’s (Evidence in Child Health to Enhance Outcomes [ECHO] KT) social media platforms (Twitter @echoktresearch, Facebook ECHO Research, and Instagram @echoktofficial) and website [27]. The overarching purpose of these interviews was to explore the concussion experience of children and parents to understand their information needs and preferences regarding concussion. For children, questions focused on the concussion experience from the child’s perspective, and for parents, questions focused on the experience of caring for a child with concussion. Interview topics included mechanism of injury, symptom experience, experience with the health care team, recovery and follow-up, and concussion information needs and preferences. The interviews were recorded and transcribed verbatim. The findings from these interviews informed the content and format of the web-based infographic. The sample demographics and full results of this qualitative study are reported by Campbell et al [28].

### Prototype Development (Phase 3)

The development of the infographic prototype involved first creating a composite infographic *skeleton*, which integrated the information needs and preferences of parents and children (as reported in the qualitative interviews) with the best available research on pediatric concussion management [28-30]. This integration determined the content for the infographic. The author AC led the development of the infographic with ongoing input and feedback from all authors. We then worked with a graphic design team that assisted in developing the accompanying artwork that would coincide with the content for the infographic.

### Parent, Youth, and Expert Feedback and Prototype Refinement

Upon completion of the infographic prototype, it was thoroughly reviewed and vetted for content accuracy by the author VP (content expert). The author VP is ideally suited for this assessment as she is well abreast of the best available research evidence in this specialized field. All authors provided extensive input and feedback on each iteration of the prototype until a finalized version was agreed upon to be disseminated for user feedback and evaluation. The prototype was then shared with a group of parents from the Pediatric Parent Advisory Group. The Pediatric Parent Advisory Group is a group of parents, legal guardians, and grandparents who serve as advisers to the ECHO Research and Alberta Research Centre for Health Evidence programs (the authors SDS and LH’s research programs) by providing advice and feedback on research aimed at improving child health outcomes [31]. Finally, the prototype was informally shared with a diverse group of youth for further advice and feedback. On the basis of the recommendations and feedback from content experts, parents, and youth, changes were made to the infographic (eg, changes to colors, images, word choice, and order of information).

### Prototype Usability Evaluation and Knowledge Change

We collected a convenience sample of participants to complete our web-based surveys. Collaborating clinicians from across Canada emailed potential participants with links to the infographic, usability test, and preintervention and postintervention knowledge and confidence tests. Potential participants included any parent, legal guardian, or grandparent. Participants were required to read and understand English. We asked for assistance from clinicians with known connections to concussion clinics, as potential participants from these clinics would be best suited to assess the relevance of the infographic and are more likely to seek out similar resources. Parent participants who previously participated in the qualitative interview portion of this research were also contacted via email with links to the infographic and surveys. In addition, we recruited participants via advertisements on our research group’s social media platforms (Twitter, Facebook, and Instagram) with a link to the surveys.

A link to the infographic, usability test, and preintervention and postintervention knowledge and confidence tests was made available to all potential participants. The study description, including potential risks and benefits, was made available before beginning the survey. Consent was implied if the web-based survey was completed and submitted. Completion of the survey was completely voluntary. Participants were asked to complete a web-based survey that assessed their perceptions of the infographic using an adapted version of the validated User Experience Questionnaire (UEQ) [32,33]. We adapted the UEQ by reducing the number of items from 26 to 11 based on relevance to our infographic. The adapted UEQ contained 11 items, rated on a 10-point Likert scale ranging from 1 (least favorable answer) to 10 (most favorable answer). Participants had the opportunity to provide free-text feedback on areas that required revisions or more information.

To evaluate the knowledge of pediatric concussion, participants were asked to answer 10 true or false questions reflecting the most common misunderstandings about concussion. The true or false questions were adopted from a questionnaire developed by McKinlay et al [34]. Participants rated their level of confidence in their response to each question using a 5-point Likert scale (very sure to very unsure). After completing the baseline knowledge test, participants were to read the infographic, and knowledge questions were answered again to assess short-term knowledge changes. Participants were again asked to rate their confidence. The UEQ was also completed following postintervention knowledge and confidence testing.

Survey data were collected from May 6, 2021, to June 14, 2021, through the Canadian web-based electronic platform SimpleSurvey. SimpleSurvey is secure, protected by several firewalls and layers of security, in alignment with Canadian privacy laws. The data collected through SimpleSurvey are completely anonymous and cannot be traced back to any individual. We followed the Checklist for Reporting the Results of Internet E-Surveys.

### Data Analysis

Data were entered into SPSS (version 26; IBM Corporation) [35], and descriptive statistics (eg, frequencies), measures of central tendency, and tests of statistical significance were completed. We conducted sample size calculations based on change in overall knowledge score to achieve a power of 80% and a level of significance of 5% (2-sided) with a 1-point difference (on a 10-point scale) between preintervention and postintervention knowledge scores (correlation estimate 0.6). A total sample size of 28 participants was required.

To assess knowledge change, we statistically compared preintervention and postintervention knowledge scores overall, as well as pretest and posttest scores for each topic. To assess knowledge change in each topic, items on the knowledge questionnaire were scored as 1 for correct and 0 for incorrect. The percentage of correct answers for each item and overall was then calculated. Owing to the nonnormality of knowledge scores, the median knowledge scores for both preintervention- and postintervention questionnaires were compared using Wilcoxon signed-rank tests.

To assess change in confidence, we statistically compared preintervention and postintervention confidence scores overall, as well as pretest and posttest scores for each topic. Likert scale scores were averaged, and differences in pre- and posttest scores were compared using paired 2-tailed *t* tests. Free-text data were analyzed using content analysis [25].

## Results

### The Tool (Web-Based Interactive Infographic)

**Figure 1:**
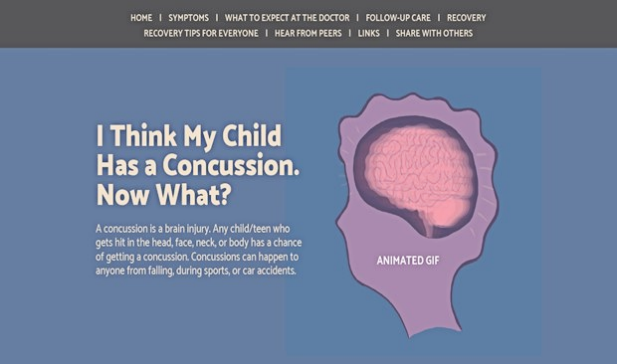
Infographic sample 1.

**Figure 2:**
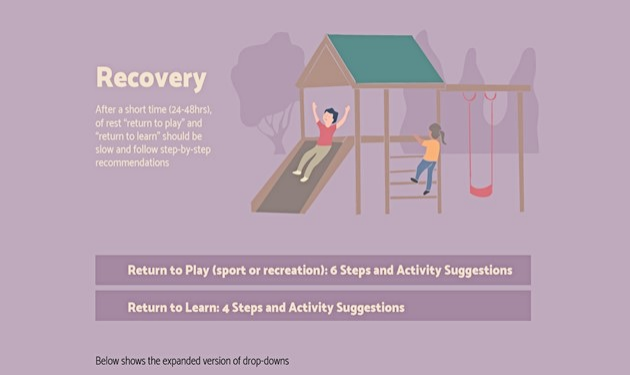
Infographic sample 2.

**Figure 3:**
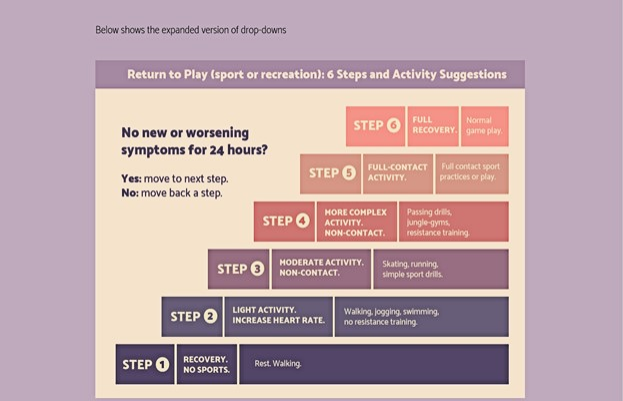
Infographic sample 3.

**Figure 4:**
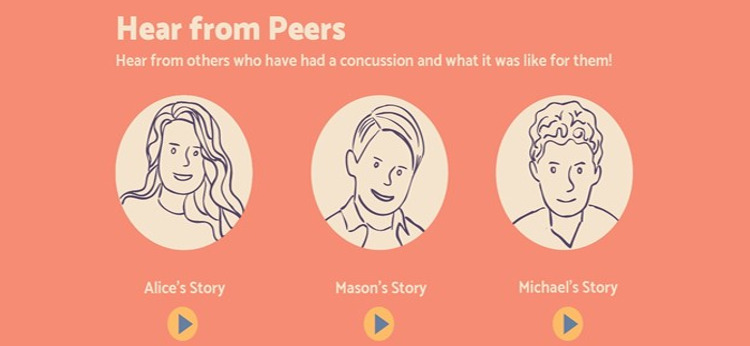
Infographic sample 4.

In collaboration with a creative design team, we developed a web-based infographic based on 14 interviews with children and parents who have had experiences with concussion. The infographic targets parents and children and was designed to incorporate information parents and children identified as both wants and needs about concussion, including symptoms, when to go to the emergency department, return to play and learn guidance, and recovery tips. Interactive elements of the infographic include an animated GIF depicting what happens to the brain inside the skull after a direct or indirect blow to the head, drop-down lists, horizontal scrolling, audio clips of children telling their story and experience with concussion, and downloadable PDF information sheets targeting teachers, coaches, and family or friends that can be shared (Figures 1-4). Modifications to the infographic were also made in careful consideration of those experiencing concussions. For example, we opted to make the font size larger than average and ensured the colors used throughout the infographic were *less bright* (softer or *dull*) to accommodate the visual disturbances and sensitivity to light often experienced with concussions [36,37]. We ensured that the characters used throughout the infographic were representative of a diverse population. On average, the infographic takes approximately 5 to 10 minutes to read and review from beginning to end.

### Usability Testing Results

In total, 43 surveys were started, of which 31 (72%) were completed. Table 1 shows the demographic characteristics of the study population.

**Table 1:** Demographic characteristics (N=31).

Variable			Value, n (%)
**Sex**			
	Female	28 (90)	
	Male	3 (10)	
**Age (years)**			
	20-29	7 (23)	
	30-39	10 (32)	
	40-49	5 (16)	
	50-59	6 (19)	
	≥60	3 (10)	
**Marital status**			
	Married, partnered, or common law	28 (90)	
	Single, separated, divorced, or widowed	2 (7)	
	Prefer not to answer	1 (3)	
**Education**			
	Postsecondary certificate or diploma	6 (19)	
	Postsecondary degree	9 (29)	
	Graduate degree	15 (48)	
	Other	1 (3)	
	Prefer not to answer	1 (3)	
**Annual household income (CAD $ [US $])**			
	40,000-59,000 (31,318-46,194)	1 (3)	
	60,000-79,000 (46,977-61,853)	1 (3)	
	80,000-99,000 (62,636-77,512)	4 (13)	
	≥100,000 (78,295)	22 (71)	
	Prefer not to answer	3 (10)	
**Number of children**			
	0	2 (7)	
	1	12 (39)	
	2	15 (48)	
	3	1 (3)	
	4	1 (3)	
**Age of children (years)**			
	<1	4 (13)	
	1-5	14 (45)	
	6-10	2 (7)	
	11-15	6 (19)	
	16-20	11 (36)	
	21-25	3 (10)	
	>25	9 (29)	
	N/A^a^	2 (7)	
**Has your child ever had a concussion?**			
	Yes	14 (45)	
	No	17 (55)	

^a^N/A: not applicable.

**Figure 5:**
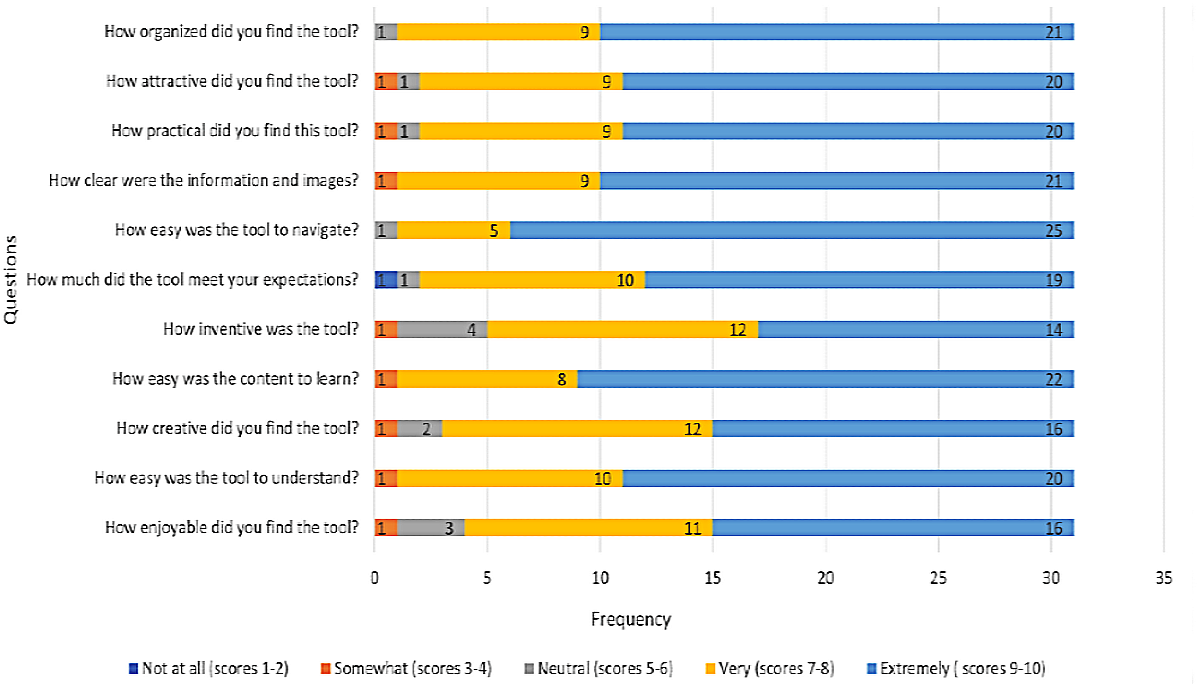
Usability testing results.

In general, parental reaction to the infographic was positive. The mean scores for each item of the usability scale ranged from 8.03 (SD 1.70) to 9.26 (SD 1.09) out of 10 (Figure 5). Only 1 parent indicated that the infographic did not meet their expectations and 1 parent commented that they would not recommend this tool to other families managing a child’s concussion. Common comments on the usability survey indicated parents felt the infographic was “simple and easy to follow,” “user friendly,” and “concise.” Other comments on the most positive aspects of the infographic included the following:

Good layout and flow to answer questions, easy to navigate, and flow chart easy to follow on next steps.

Participant 14

The points were well highlighted. Easy to follow. Informative, quick to look at for what to do or watch for. Not too overwhelming. Touched the most important information.

Participant 23

It did not overwhelm with information.

Participant 8

Some parents felt that the infographic colors were “dull” and “sometimes hard to view” (ie, Participants 26 and 29). For instance, a parent said:

Mostly blocks of words, not unlike a pamphlet. Colours somewhat dull and lacking contrast between text and background. May be difficult for people with low vision or differences in colour perception.

Participant 11

### Knowledge Evaluation and Confidence in Response

The median preintervention knowledge score across the 10 knowledge topics was 9 (IQR 9-10), and the median postintervention knowledge score was 9 (IQR 9-10). A Wilcoxon signed-rank test showed no statistically significant difference in median knowledge scores before and after viewing the infographic (*Z*=−0.593; *P=*.53). Individually, an overall knowledge gain (although minor) was observed for topics 2, 7, and 10, and an overall knowledge loss was observed for topics 1, 6, 8, and 9, but Wilcoxon signed-rank tests showed no statistically significant changes before and after the intervention (Table 2). In the remaining 3 topics (topics 3, 4, and 5), 100% (31/31) of the parents successfully identified the correct response before viewing the infographic, and this was retained after.

The mean preintervention confidence score across the 10 knowledge topics was 3.9 (SD 0.56; 3.9/5, 78%) and the mean postintervention confidence score was 4.4 (SD 0.44; 4.4/5, 88%). A paired 2-tailed *t* test showed a statistically significant difference in average confidence scores before and after viewing the infographic (t_30_=−5.083; *P*<.001). Across each knowledge topic, paired 2-tailed *t* tests showed a statistically significant difference in confidence in answering the true or false questions for 70% (7/10) of the topics (topics 2, 3, 5, 6, 7, 9, and 10; Table 3).

**Table 2:** Changes in knowledge before and after exposure to the intervention.

Question (correct answer) and answer combination			Scenario				Frequency, n (%)		Before exposure, % correct			After exposure, % correct			*P* value	
			Before exposure		After exposure											
**A concussion only occurs when there is loss of consciousness (F^a^)**										100			97			.32
	Combination 1	T^b^		F		0 (0)										
	Combination 2	F		T		1 (3)										
	Combination 3	T		T		0 (0)										
	Combination 4	F		F		30 (97)										
**A concussion only occurs after a blow directly to the head (F)**										87			97			.32
	Combination 1	T		F		4 (13)										
	Combination 2	F		T		1 (3)										
	Combination 3	T		T		0 (0)										
	Combination 4	F		F		26 (84)										
**Confusion is not a sign of concussion if it clears within 5 minutes (F)**										100			100			>.99
	Combination 1	T		F		0 (0)										
	Combination 2	F		T		0 (0)										
	Combination 3	T		T		0 (0)										
	Combination 4	F		F		31 (100)										
**It is safe to return to playing sports as soon as the child is no longer confused (F)**										100			100			>.99
	Combination 1	T		F		0 (0)										
	Combination 2	F		T		0 (0)										
	Combination 3	T		T		0 (0)										
	Combination 4	F		F		31 (100)										
**Concussion symptoms are only apparent at the time of injury (F)**										100			100			>.99
	Combination 1	T		F		0 (0)										
	Combination 2	F		T		0 (0)										
	Combination 3	T		T		0 (0)										
	Combination 4	F		F		31 (100)										
**Being *knocked out* is not the same as a concussion (T)**										77			65			.16
	Combination 1	T		F		6 (19)										
	Combination 2	F		T		2 (7)										
	Combination 3	T		T		18 (58)										
	Combination 4	F		F		5 (16)										
**Someone with a concussion should be kept awake for the first 24 hours after injury (F)**										90			94			.32
	Combination 1	T		F		1 (3)										
	Combination 2	F		T		0 (0)										
	Combination 3	T		T		2 (7)										
	Combination 4	F		F		28 (90)										
**A concussion does not have longer-term effects (F)**										97			94			.32
	Combination 1	T		F		0 (0)										
	Combination 2	F		T		1 (3)										
	Combination 3	T		T		1 (3)										
	Combination 4	F		F		29 (94)										
**Children will recover better from a concussion than adults (F)**										61			58			.56
	Combination 1	T		F		1 (3)										
	Combination 2	F		T		2 (7)										
	Combination 3	T		T		11 (36)										
	Combination 4	F		F		17 (55)										
**Sometimes, concussion symptoms can take hours or days to show up (T)**										90			97			.32
	Combination 1	T		F		1 (3)										
	Combination 2	F		T		3 (10)										
	Combination 3	T		T		27 (87)										
	Combination 4	F		F		0 (90)										

^a^F: false.

^b^T: true.

**Table 3:** Confidence in knowledge responses (N=31).

Question (correct answer) and pre- or posttest			Confidence, n (%)											Score, mean (SD)			*P* value	
			Not at all confident		Somewhat confident		More than somewhat but not very confident		Very confident		Extremely confident							
**A concussion only occurs when there is loss of consciousness (false)**																		.06
	Pretest	0 (0)		0 (0)		2 (7)		9 (29)		20 (65)		4.58 (0.62)						
	Posttest	0 (0)		0 (0)		1 (3)		6 (19)		24 (77)		4.74 (0.51)						
**A concussion only occurs after a blow directly to the head (false)**																		.02^a^
	Pretest	1 (3)		1 (3)		5 (16)		9 (29)		15 (48)		4.16 (1.04)						
	Posttest	0 (0)		0 (0)		2 (7)		7 (23)		22 (71)		4.65 (0.61)						
**Confusion is not a sign of concussion if it clears within 5 minutes (false)**																		.004^a^
	Pretest	0 (0)		1 (3)		12 (39)		10 (32)		8 (26)		3.81 (0.87)						
	Posttest	0 (0)		1 (3)		3 (10)		8 (26)		19 (61)		4.45 (0.81)						
**It is safe to return to playing sports as soon as the child is no longer confused (false)**																		.06
	Pretest	0 (0)		10 (32)		5 (16)		0 (0)		16 (52)		4.35 (0.76)						
	Posttest	0 (0)		1 (3)		0 (0)		8 (29)		22 (71)		4.65 (0.66)						
**Concussion symptoms are only apparent at the time of injury (false)**																		.03^a^
	Pretest	0 (0)		0 (0)		4 (13)		11 (36)		16 (52)		4.39 (0.72)						
	Posttest	0 (0)		0 (0)		1 (3)		7 (23)		23 (74)		4.71 (0.53)						
**Being *knocked out* is not the same as a concussion (true)**																		.008^a^
	Pretest	0 (0)		2 (7)		14 (45)		10 (32)		5 (16)		3.58 (0.85)						
	Posttest	0 (0)		2 (7)		6 (19)		12 (39)		11 (36)		4.03 (0.91)						
**Someone with a concussion should be kept awake for the first 24 hour after injury (false)**																		.001^a^
	Pretest	1 (3)		4 (13)		10 (32)		10 (32)		6 (19)		3.52 (1.06)						
	Posttest	1 (3)		1 (3)		3 (10)		9 (29)		17 (55)		4.29 (1.01)						
**A concussion does not have longer-term effects (false)**																		.07
	Pretest	0 (0)		0 (0)		5 (16)		14 (45)		12 (39)		4.23 (0.72)						
	Posttest	0 (0)		0 (0)		3 (10)		9 (29)		19 (61)		4.52 (0.68)						
**Children will recover better from a concussion than adults (false)**																		.004^a^
	Pretest	3 (10)		6 (19)		14 (45)		5 (16)		3 (10)		2.97 (1.08)						
	Posttest	2 (7)		1 (3)		11 (36)		12 (39)		5 (16)		3.55 (1.03)						
**Sometimes, concussion symptoms can take hours or days to show up (true)**																		<.001^a^
	Pretest	2 (7)		1 (3)		8 (26)		12 (39)		8 (26)		3.74 (1.09)						
	Posttest	0 (0)		0 (0)		3 (10)		11 (36)		17 (55)		4.45 (0.68)						

^a^Statistically significant at *P*<.05.

## Discussion

### Principal Findings

This study evaluated the usability of a novel, web-based infographic for parents who have experiences managing a child with a concussion, in addition to parental knowledge about pediatric concussion and confidence in their responses. Previous research has explored student and parent knowledge and perceived confidence about brain injury and concussion but has not assessed changes in knowledge or confidence before and after exposure to an educational intervention [38]. Our study is the first to examine how web-based and arts-based media impact parental knowledge and confidence about pediatric concussions.

Parental concussion education is critically important, as many concussion signs and symptoms may not appear until hours or even days after the incident. As such, the onus falls on parents to identify signs and symptoms to ensure prompt and proper diagnosis, treatment, management, and safe return to play or school [9]. Previous studies on parental concussion knowledge have found that parents are generally knowledgeable about concussion signs, symptoms, and recovery time even before exposure to an educational intervention, with small improvements in knowledge following exposure [9,13]. Parents in our study were also found to be generally knowledgeable about concussion before exposure to our infographic. Overall, the results of this study showed no significant overall increase in concussion knowledge. This may be because most parents in our study had previous experience with concussion and possessed high levels of concussion knowledge at baseline. A more diverse sample (particularly those without previous concussion experience) and more pronounced knowledge deficits at baseline may have yielded more significant knowledge changes.

Unique to our study was the evaluation of not only parental knowledge changes but also changes in confidence in their responses. After exposure to the infographic, parents’ confidence in their responses significantly increased in 70% (7/10) of the content areas. This suggests that our infographic was effective in helping parents feel more confident about their knowledge of pediatric concussions. Increasing parental confidence in knowledge of their child’s illness or injury may positively influence their child health care decision-making (eg, when seeking medical attention, home care, and recovery), ultimately improving child health outcomes [15]. This phenomenon was observed in a previous study conducted by our research team in the area of pediatric chronic pain [22].

Overall, the results on the usability of the infographic were positive, with most parents rating each aspect of the tool as very favorable or extremely favorable. Our study and others have demonstrated that innovative media, using narrative and artistic elements, is a promising approach for communicating complex health information to parents and families [18,19,22]. Although time and resource strain often determine how resources are developed, more innovative media may be beneficial in evoking relevance, timely accessibility, and engagement. In fact, more innovative media such as cartoons and videos have been found to be superior to standard medical sheets in transferring information to consumers [7]. Our chronic pain study yielded results similar to those in this study, suggesting that innovative, web-based, and arts-based interventions (eg, e-books and infographics) are viewed positively by parents and may increase parental confidence in their knowledge of pediatric conditions [22].

The systematic review by Feiss et al [9] found only 3 concussion education programs to be evaluated in the literature. Furthermore, although these programs include written content directed toward parents, there are no programs specifically designed for parents. Our infographic is unique in that it was designed specifically for parents based on their information needs and preferences, and parents were involved throughout the tool development process. Furthermore, our infographic was designed to be *safe* or *comfortable* for youth with concussions to view, as we carefully considered elements such as font size and colors that would not cause eye strain and potentially exacerbate concussion symptoms. Although the rapidly developing evidence base in pediatric concussion places increased demands on updating information that is included in educational interventions to ensure they are timely and relevant, researchers have an opportunity to expand on the evidence being shared by taking a more participatory approach, involving end users in the development of these interventions.

The finalized version of this infographic (finalized February 2022) is now being disseminated through established social media platforms, including ECHO Research’s Instagram, Twitter, Facebook, and website [27] and Translating Emergency Knowledge For Kids [39], which is a national network of health professionals and parents whose goals are to improve emergency care for children.

### Limitations

We relied on parent self-report data. The parents in our study consistently possessed high levels of education, and our findings cannot be extrapolated to all parents, including those with more pronounced information deficits or poor health literacy. Recruitment and data collection for this study occurred during the COVID-19 pandemic; thus, in adherence to government guidelines, recruitment occurred on the web through convenience sampling, which may not have provided the most information-rich sources. We acknowledge the approximately 25% (31/43) dropout attrition in our surveys. The reasons for dropout attrition in our study are unknown, although they are common in web-based surveys. However, careful review of the survey is warranted to potentially diminish dropout attrition in the future. Our study evaluated short-term changes in knowledge and confidence. Future research should examine changes in knowledge and confidence over time, as well as whether this change affects decisions made regarding the care and outcomes of the child. In addition, approximately half of the parents in our study had previous experience with concussions and may have received information about concussions via other sources. This may confound our results in terms of the effectiveness of the infographic on knowledge and confidence. Furthermore, the baseline knowledge score was very high, leaving little room to see an effect in terms of knowledge change. Our study did not ask about previous education sources, training, or exposure to pediatric concussions. A better understanding of the impact of various forms of education on knowledge and confidence, including participants with lower socioeconomic backgrounds, will help improve the design and format of future educational interventions.

### Conclusions

Our results demonstrated that parents positively rated a web-based infographic about pediatric concussion, and this infographic increased their confidence in knowledge of pediatric concussion. These findings hold promise for future development, application, and effectiveness testing of web-based, arts-based KT interventions for transferring complex health information to parents. Future research using innovative digital media for knowledge transfer with different clinical conditions and participant demographics (ie, children and different parents) should be explored as well as the effectiveness of different formats (eg, videos, e-books, and standard information sheets).

## Abbreviations

ECHO: Evidence in Child Health to Enhance Outcomes
KT: knowledge translation
UEQ: User Experience Questionnaire
